# The potential of organoids in renal cell carcinoma research

**DOI:** 10.1186/s12894-024-01511-x

**Published:** 2024-06-11

**Authors:** Qiuyang Chen, Xuan Sun, Yubei Li, Xinyue Yang, Xuejian Yang, Haifei Xu, Hongzhou Cai, Jun Hu

**Affiliations:** 1 Department of Urology, Jiangsu Cancer Hospital & The Affiliated Cancer Hospital of Nanjing Medical University & Jiangsu Institute of Cancer Research, Nanjing, China; 2 Department of Radiology, The Fourth School of Clinical Medicine, Nanjing Medical University, Nanjing, Jiangsu China; 3 Department of Urology, Suqian First Hospital, Suqian, China; 4 Department of Urology, Nantong Tumor Hospital, Nantong, China; 5 Department of Nursing, Jiangsu Cancer Hospital & The Affiliated Cancer Hospital of Nanjing Medical University & Jiangsu Institute of Cancer Research, Nanjing, China

**Keywords:** Organoid, Renal cell carcinoma, Precision medicine, 3D tumor models

## Abstract

Renal cell carcinoma, a leading cause of death in urological malignancies, arises from the nephron. Its characteristics include diversity in disease biology, varied clinical behaviors, different prognoses, and diverse responses to systemic therapies. The term ‘organoids’ is used to describe structures resembling tissues created through the three-dimensional cultivation of stem cells in vitro. These organoids, when derived from tumor tissues, can retain the diversity of the primary tumor, mirror its spatial tissue structure, and replicate similar organ-like functions. In contrast to conventional two-dimensional cell cultures and the transplantation of tumor tissues into other organisms, organoids derived from tumors maintain the complexity and microenvironment of the original tumor tissue. This fidelity makes them a more reliable model for the development of cancer drugs, potentially accelerating the translation of these drugs to clinical use and facilitating personalized treatment options for patients. This review aims to summarize the recent advancements in the use of organoids for studying renal cell carcinoma, focusing on their cultivation, potential applications, and inherent limitations.

## Introduction

The incidence of renal cell carcinoma (RCC) has been on an upward trajectory in recent years, prompting an intensified focus on its therapeutic approaches. RCC constitutes about 3% of all cancer types [1]. As the predominant solid mass in the kidney, RCC comprises nearly 90% of all renal malignancies. The occurrence of kidney cancer shows a gender disparity, with men being more affected than women at a ratio of approximately 1.5:1. Predominantly, RCC is most prevalent in the elderly, especially those aged between 60 and 70 years. Lifestyle factors such as smoking, obesity, and hypertension have a significant correlation with the etiology of RCC. Moreover, a higher risk of RCC is noted in individuals with a first-degree relative diagnosed with the disease [2].

In 2016, the World Health Organization’s classification system comprehensively detailed RCC, categorizing it into various types, including both aggressive and less aggressive forms. These types include clear cell renal cell carcinoma (ccRCC), multilocular cystic renal neoplasm with low malignant potential, papillary renal cell carcinoma (pRCC), fumarate-hydratase deficient renal cell carcinoma (fhRCC), chromophobe renal cell carcinoma (chRCC), collecting duct carcinoma (CDC), renal medullary carcinoma (RMC), MiT family translocation renal cell carcinomas (tRCC), succinate dehydrogenase-deficient renal cell carcinoma (SDDRCC), mucinous tubular and spindle cell carcinoma, tubulocystic renal cell carcinoma, acquired cystic disease-associated renal cell carcinoma, clear cell papillary renal cell carcinoma, unclassified renal cell carcinoma (uRCC), papillary adenoma, and oncocytoma, among others [3]. The array of RCC subtypes exhibit distinct histological and clinical characteristics, posing challenges in prognosis determination and therapy response prediction [4]. Consequently, the development of precise and personalized treatments for RCC patients is becoming increasingly imperative. The burgeoning field of organoid research offers novel avenues for exploring RCC pathogenesis and experimenting with targeted treatment strategies.

Organoids, created using adult or pluripotent stem cells, are three-dimensional (3D) cultured tissue analogs exhibiting specific spatial structures. They serve as effective intermediaries bridging the gap between cellular and tissue-level studies [5]. In tumor research, organoids offer a robust model capable of being cultured, passaged, cryopreserved, and revived over extended periods. They maintain structural and functional similarity to the original tissue [6]. Unlike traditional two-dimensional (2D) cultures and patient-derived xenografts (PDX), tumor organoids demonstrate a notably higher success rate in construction. They can be cultured for extended durations at reduced costs, facilitating genetic modifications and large-scale drug testing [7]. Moreover, 3D-culture methods preserve the histological characteristics of tumors providing a more realistic environment for study. The development of tumor organoids alongside their healthy tissue counterparts enables the screening of drugs that are effective against tumors but less harmful to healthy tissues. This advancement paves the way for the clinical application of individualized tumor treatments [8].

This article presents a comprehensive review, synthesizing the latest advancements in the field of RCC research using organoids. It encompasses a detailed analysis of the cultivation methodologies, various applications, and the inherent limitations of organoids in the context of RCC.

## Culture methodologies

The primary method for organoid culture is 3D cell culture, where stem cells are cultured in a matrix gel with various inhibitors/activators and cytokines. Research is currently focusing on advancing milli-fluidic chip culture and suspension culture, which are dependent on microfluidic devices.

**Fig. 1:**
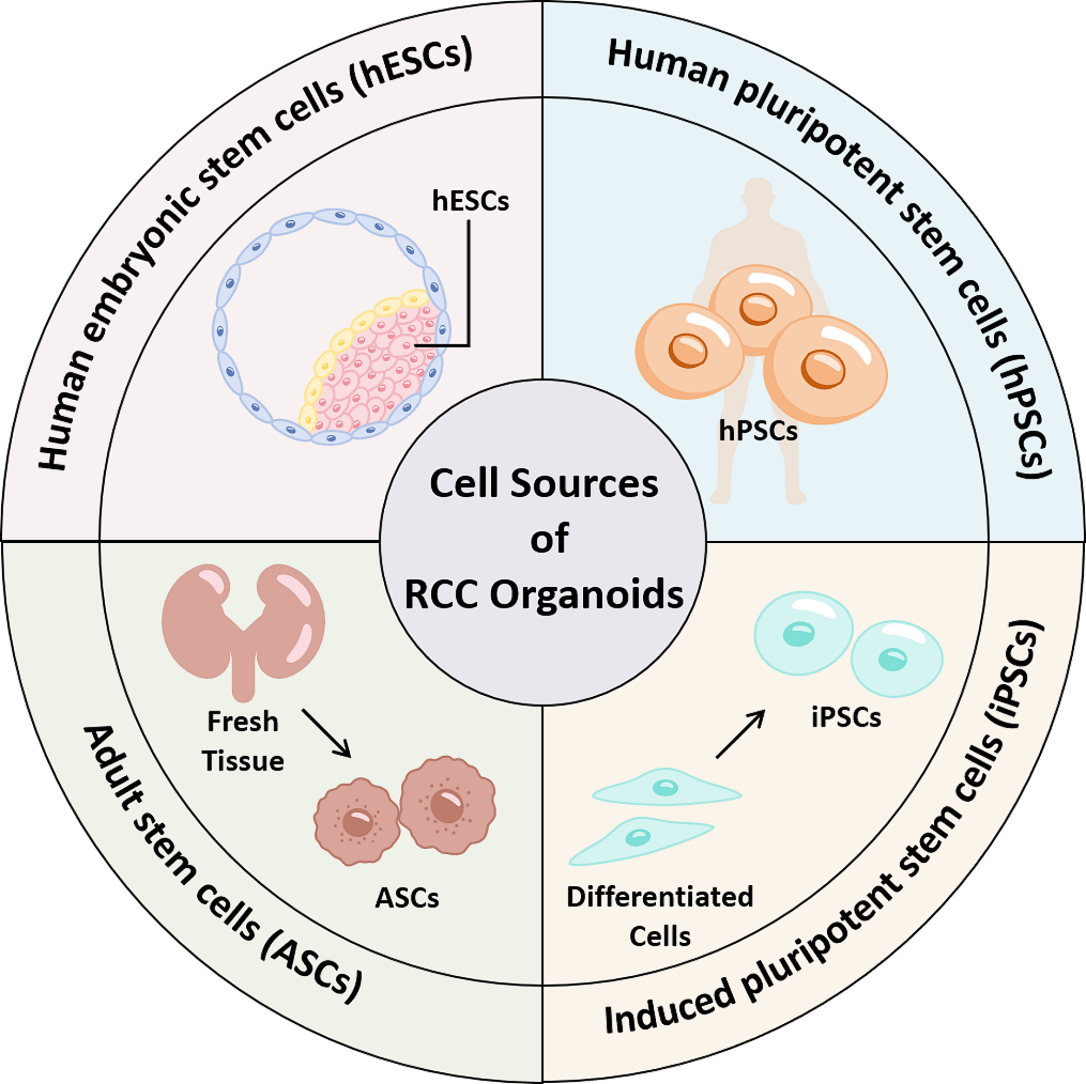
This figure summarizes the typical cell types used in organoid cultures, including hESCs, hPSCs, iPSCs, and ASCs

The upcoming sections of this article will delineate the frequently used materials and methodologies in organoid culture. This includes an overview of the typical cell types employed in organoid cultures (Fig. 1) and the development of cell culture systems. And a correct and detailed understanding of the culture methods for normal renal organoids is an important prerequisite for RCC organoid culture methods discussed in this article. Making appropriate modifications in the culture of normal organoids can accurately replicate the tumor microenvironment and preserve the genetic and phenotypic characteristics of RCC.

### Cell sources

#### Human embryonic stem cells (hESCs)

hESCs, undifferentiated cells derived from the inner cell mass or primordial gonads of early embryos, have the capacity to differentiate into all three primary germ layers of the embryo, leading to the formation of various cell types under specific conditions.

There are many methods to culture organoids from hESCs, including two distinct protocols that have been established for the differentiation of primitive somite (PS) and intermediate mesoderm (IM) into metanephric mesenchyme (MM) and ureteric bud (UB) [9].

The differentiation process involved culturing cells in a medium infused with Vitronectin or Matrigel, which simulates the properties of natural connective tissue. In the first protocol, the medium was supplemented with activin A, bone morphogenetic protein (BMP) 4/7, fibroblast growth factor (FGF) 9, and retinoic acid (RA). The second approach involved the addition of the Wnt agonist CHIR99021 along with FGF9.

#### Human pluripotent stem cells (hPSCs)

**Fig. 2:**
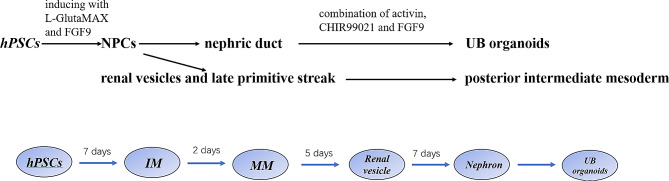
This figure summarizes the protocol using L-GlutaMAX, FGF9 and CHIR99021 that culture organoids from hPSCs and temporal stages of the organoid development of renal organoids

hPSCs possess remarkable self-renewal capabilities and the potential to differentiate into a wide range of somatic cell types and tissues, making them highly valuable for disease modeling and regenerative applications. A highly efficient and serum-free process for differentiating hPSCs into ureteric bud (UB) organoids and functional collecting duct (CD) cells involves several steps with L-GlutaMAX, FGF9 and CHIR99021 (Fig. 2). Initially, hPSCs are induced to form pronephric progenitor cells (NPCs),, which then aggregate into spheres resembling the nephric duct. In a 3D matrix, these spheres evolve into UB organoids, displaying branching morphogenesis [10]. In another approach, hPSCs are cultured in a ReproFF2 medium forming well-defined circular colonies. These undifferentiated hPSCs are isolated and passaged every seven days. For inducing NPCs, Advanced RPMI 1640 medium with L-GlutaMAX and FGF9 is employed, followed by the addition of CHIR99021 to generate structures akin to renal vesicles [11]. A successful method selectively induces metanephric NPCs, which can develop into in vivo counterparts. The combination of activin, GSK-3β inhibitor CHIR99021, and FGF9 further directs NPCs to form renal vesicles and late primitive streak, eventually giving rise to posterior intermediate mesoderm. These structures can self-organize into nephronic structures, expressing markers of podocytes, proximal tubules, Henle’s loops, and distal tubules, arranged in a continuous, organized manner [12].

The efficiency of these differentiation methods is influenced by the inherent variability of hPSC lines because the cells in the interstitial space of kidney organoids are not yet fully characterized.

#### Induced pluripotent stem cells (iPSCs)

iPSCs, the primary source for organoid formation, are derived by reprogramming differentiated cells from patients or healthy individuals. Organoids created from iPSCs can model hereditary or infectious kidney diseases, making iPSCs a key resource for organoid culture.

Taguchi and Nishinakamura [13] determined that a combination of retinoic acid (RA), CHIR99021 at a low concentration (1 µM), and FGF9 (5 ng/mL), either with or without glial cell line-derived neurotrophic factor (GDNF), an attractant similar to Wnt11, is optimal for inducing differentiation. This mix promotes the anterior intermediate mesoderm (AIM) from mouse or human iPSCs to undergo a mesenchymal-to-epithelial transition (MET), leading to the formation of the epithelialized Wolffian duct (WD). Ultimately, this process results in the development of the UB with the ability to branch.

Musah’s team developed a method to differentiate human iPSCs into mesoderm cells using activin A, CHIR99021, and Rho kinase (ROCK) inhibitor Y27632. Subsequently, BMP7 and CHIR99021 were used to induce the formation of intermediate mesoderm cells. These cells were then cultured in a medium containing BMP7, activin A, vascular endothelial growth factor (VEGF), retinoic acid (RA), and CHIR99021 for 4 to 5 days to successfully generate podocytes [14].

Controllable 3D growth environments are crucial for enhancing the consistency and maturity of organoids. Consequently, iPSC-derived kidney organoids were matured in fully synthetic self-assembling peptide hydrogels (SAPHs) with varying degrees of stiffness. This minimally complex microenvironment is tailored for kidney differentiation. As a result, organoids with high viability were produced, although a slight decrease in cell viability was observed with increased matrix stiffness [15].

#### Adult stem cells (ASCs)

ASCs, found in differentiated tissues, have the ability to self-renew and differentiate. Fresh tissue from patients with clear cell RCC was used to develop air-liquid interface (ALI) patient-derived organoids (PDOs). This method resulted in organoids that resembled the tumor histology and immune microenvironment [16, 17]. Organoid growth was initially achieved with fetal calf serum and enhanced by adding growth factors like WNT3A, EGF, NOGGIN, and RSPO1 (WENR). Passaging of ALI PDOs was performed by addition of 200 units collagenase IV for 30 min at 37 °C until the collagen was dissociated. Then, three washes with PBS and EDTA were performed to inhibit collagenase activity. The ALI PDOs were absorbed with 1 ml of type I collagen solution and replicated into a new ALI collagen gel at the desired mass density. These ALI PDOs, with a solid growth pattern, remained viable for over 30 days in culture.

Another approach mixed tumor cells with a cold basement membrane extract containing EGF. In the study of Li et al. [18], organoids were passaged every 2–3 weeks at a split ratio of 1:2–1:3, leading to the generation of renal cancer organoid lines such as ccRCC, pRCC, and chRCC.

In another protocol, cells were also maintained in a serum-free medium with EGF and basic fibroblast growth factor (bFGF). They were then cultured in a medium with low-density Matrigel, EGF, bFGF, ROCK inhibitor, and A8301, an inhibitor of TGF-β, ALK4, and ALK7 [19].

Organoids from ASCs have a short cultivation cycle and high genetic stability but lack interstitial and endothelial cells, marking them as primarily epithelial cell systems.

#### Urine-derived stem cells (USCs)

Compared to adult somatic stem cells and human pluripotent stem cells, urine-derived stem cells (USCs) can be obtained through a non-invasive method, and have been proven to possess regenerative properties. They exhibit robust proliferative potential and can successfully differentiate into urothelial lineages. Organoids differentiated from USCs have been applied in research on hereditary kidney diseases and motor neuron diseases [20].

The study by Liang Chen et al. [21] indicates that USC-organoids share similar morphological, histological, gene expression profiles, and nephrotoxicity screening capabilities with kidney organoids derived from other sources. Research by Wan et al. [20] also notes that urothelial organoids produced from stem cells isolated from urine resemble natural urothelial organoids in phenotype and function. In summary, USC-organoids not only face fewer ethical restrictions but also possess comprehensive scientific value. Although the collection process may be more time-consuming compared to adult somatic stem cells and human pluripotent stem cells, the non-invasive advantage makes them a promising adjunct for personalized treatment of renal cancer patients in future clinical applications.

### Establishment of cell culture system

**Fig. 3:**
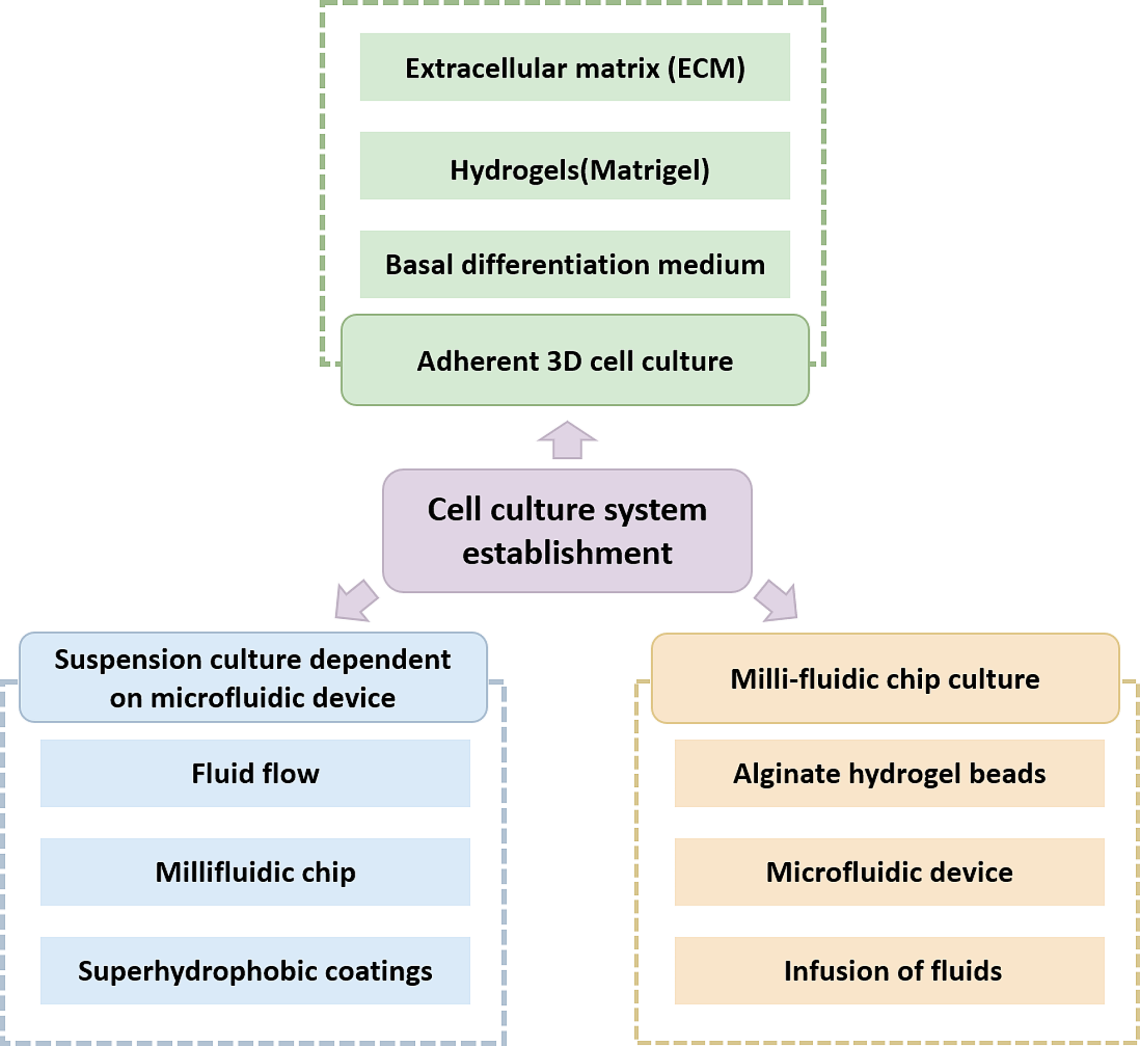
This figure focuses on a variety of organoid culture systems, including adherent 3D cell cultures, milli-microfluidic chip cultures, and suspension cultures dependent on microfluidic devices

Traditional media used for cultivating cancer organoids generally include components such as B-27 supplement, nicotinamide, R-spondin1, noggin, N-acetyl-l-cysteine, A83-01, SB202190, FGF 10, EGF, and Y-27,632 [22, 23]. Building on this foundation, a variety of innovative cultivation systems have been developed, encompassing adherent 3D cell culture, milli-fluidic chip culture, and suspension culture reliant on microfluidic devices (Fig. 3).

#### Adherent 3D cell culture

3D multicellular miniature organoids are extensively used for mimicking organ development and disease progression. The extracellular matrix (ECM) plays a vital role in the self-renewal and differentiation of stem cells by providing a necessary scaffold for cell adhesion and growth during organoid culture. Hydrogels, particularly Matrigel, are frequently utilized as scaffolds to support cell proliferation in organoid cultures, facilitating the formation of complex structures that resemble corresponding organs and preserve their physiological structure and functional traits.

In Low et al. [24], cells were dissociated using Accutase and then aggregated in a basal differentiation medium. This process initially generated epiblast spheroids, which subsequently developed into kidney tubules. After cell dissociation, the cells, when sandwiched between two layers of diluted Matrigel, formed compact, ball-like colonies and then developed internal cavities [25].

#### Milli-fluidic chip culture

Renal organoids generated from hPSCs feature sections resembling glomeruli and tubules, but these components often remain underdeveloped in static cultures. The application of microfluidics technology significantly addresses this challenge. By cultivating kidney organoids in an environment with fluid flow and vascularization, the development of nephron structures are notably enhanced.

An in vitro technique for culturing kidney organoids on a millifluidic chip significantly enhances the expansion of their intrinsic endothelial progenitor cells and fosters the development of vascular networks with perfusable lumens encircled by mural cells. The millifluidic chip is characterized by its high flux and small volume, enabling sample and reagent conservation. It also facilitates real-time monitoring and manipulation. The application of superhydrophobic coatings on the chip’s surface creates distinct physical separations between each micropore, ensuring each one has its own isolated liquid environment. This design simplifies the addition and alteration of reagents.

In comparison to static cultures, vascularized renal organoids cultured under flow conditions exhibited more advanced development in their podocyte and tubular compartments, which is required to facilitate functional morphogenesis of podocytes in vitro. This advancement was characterized by enhanced cellular polarity and a more mature adult gene expression profile [26].

#### Suspension culture dependent on microfluidic device

Microfluidic techniques, known for their high precision, reduced contamination risk, and user-friendly operation, commonly utilize cell-laden alginate hydrogel beads. These beads are biologically inert and are used in suspension cultures through various methods such as microplate, pendant drop, rotational culture, and magnetic suspension.

Human renal cancer cells were incorporated into alginate hydrogels using a straightforward and cost-effective microfluidic device which was designed in a computer-aided design software as a three-layer structure. The top, middle, and bottom layers show the inlet and outlet holes, the outline of the fluidic channel, and the lower boundary of the microfluidic device in two dimensions, respectively. This device facilitates the infusion of various fluids, including cell solutions, alginate, calcium chloride, and mineral oil, to produce water-in-oil droplets for size-controllable organoid culture [27]. This technique enables the rapid and economical creation of uniformly sized organoids, suitable for replicable experimental setups.

## Applications

**Fig. 4:**
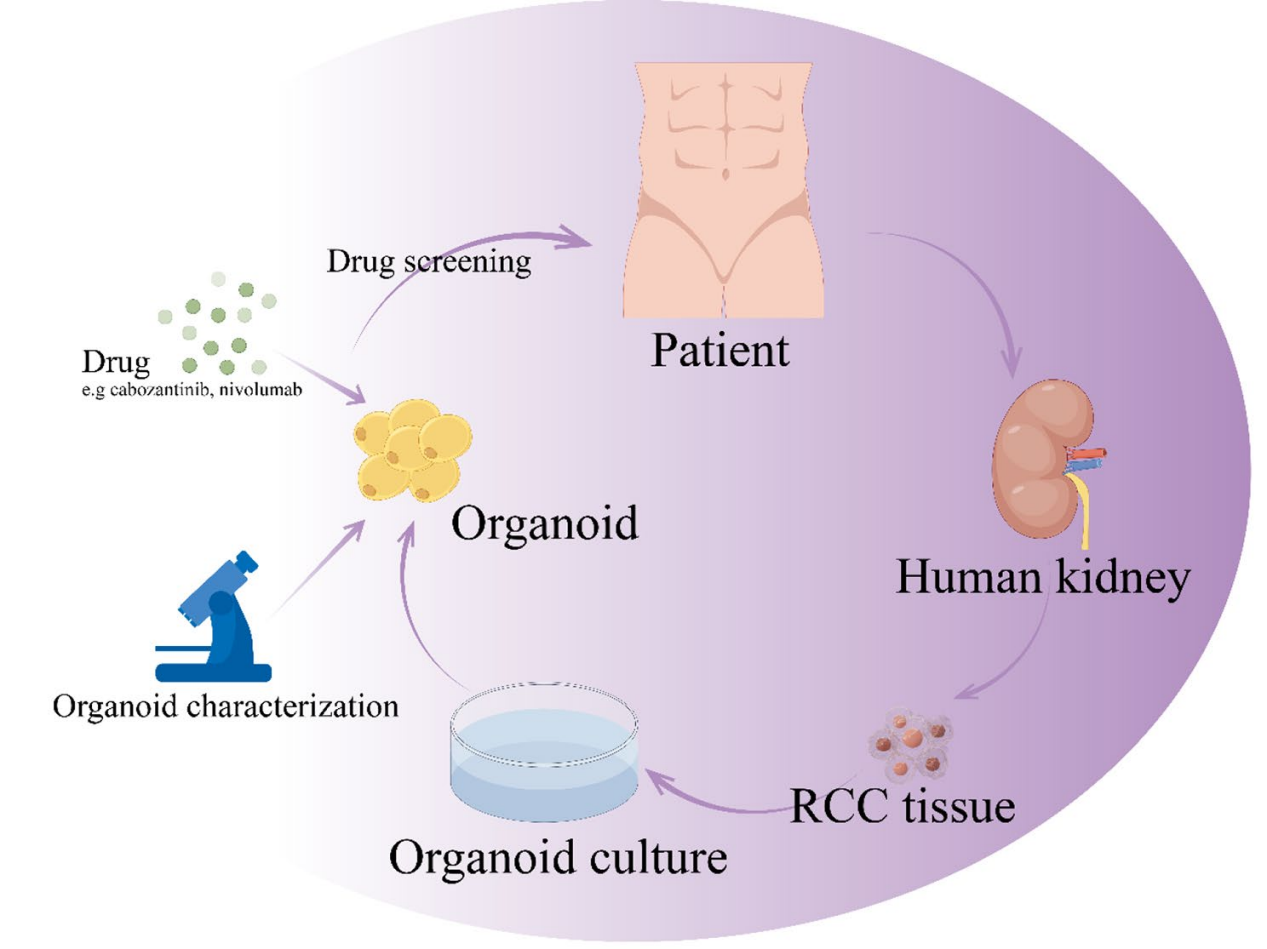
This figure shows the application of organoids for RCC. With the ability to mimic the structure and function of organs, organoids offer a personalized and efficient approach to studying the pathological mechanism, testing potential treatments, and developing new therapies for RCC

Organoids have become a valuable tool in RCC research. Organoids provide a tailored and effective means for exploring the pathophysiological mechanisms of RCC, assessing prospective treatments, and formulating novel therapeutic strategies (Fig. 4).

### Exploring the pathophysiological mechanisms of RCC

Previous RCC research primarily utilized 2D cell cultures and animal models. While 2D cultures, like human renal tubular epithelial cells, offer ease of operation and are conducive to gene editing and high-throughput screening, they present limitations. These include a single-layered cell structure, absence of vascularization and immune systems, restricting their capability to accurately replicate human kidney physiology and pathology [28]. Animal models, despite simulating human disease pathophysiology, cannot entirely represent human kidney genetics due to their non-human tissue origin.

Human kidney organoids serve as an intermediary between traditional 2D cultures and animal models, combining experimental ease with a comprehensive simulation of human genetic traits. The incorporation of CRISPR/Cas9-mediated gene editing in these organoids allows for an in-depth study of gene functions. They offer significant enhancements to classical models for constructing in vitro RCC models and investigating pathogenesis [29].

A study utilized hiPSCs from pRCC patients to create an in vitro model for hereditary renal cancer with c-met mutations. These hiPSCs were differentiated into 3D renal organoids, exhibiting features like glomeruli and proximal tubules, and expressing markers associated with pRCC, renal progenitor cells, and endothelial cells. The gene expression in these organoids closely matched that observed in a large group of pRCC patients, making this model valuable for understanding inherited kidney cancer and aiding in the development of targeted therapies [30]. In another research, high expression of piR-1742 in RCC tumors was linked to poorer patient prognosis. Experiments with RCC organoid models confirmed that inhibiting piR-1742 significantly reduced tumor growth [31]. Jual et al. [32] also found that the overexpression of SATB2 in RCC samples was associated with poor prognosis. Targeting SATB2 or BRD7 in patient-derived organoids with high YAP expression effectively inhibited tumor proliferation, highlighting organoids’ role in studying and demonstrating RCC pathogenesis.

Recent progress in the field has been encouraging, yet it remains essential to recognize that the development and use of renal cancer-derived organoids are in early stages. Enhancing the methodologies for these organoids is necessary to broaden their potential in both research and clinical applications [33]. Concurrently, extensive research is being conducted to deepen understanding of renal cell carcinoma’s molecular dynamics and to develop more effective therapeutic strategies.

### Anticancer drug development

In laboratory settings, tumor drug development initially involves evaluating the effects of treatments on cancer cell proliferation in 2D in vitro models, a process crucial for screening a broad spectrum of new anti-tumor agents. Once potential anti-tumor drugs are identified, research typically progresses to Patient-Derived Xenograft (PDX) models for in vivo efficacy and safety evaluation [34, 35]. Remarkably, about 85% of drugs in the preclinical stage do not pass clinical trials, despite promising results in initial screenings, mainly due to unsatisfactory safety and efficacy profiles. This statistic underscores the limitations of traditional drug screening models for tumors. Specifically, 2D tumor cell cultures often fail to preserve the original tumor’s genetic characteristics and microenvironment when cultured under selective conditions [36]. According to Dhimolea et al. [37], 2D cultured tumor cells demonstrate less resistance to drugs compared to tumor organoids. These cells are more prone to apoptosis under the same chemotherapy dosage and have lower survival rates under equivalent treatment durations. This observation indicates that the 3D structure of organoids significantly influences in vitro tumor drug sensitivity assessments. While PDX models, which mimic the 3D structure of tumors, are more representative, their extended building and analysis periods, and unsuitability for high-throughput screening, highlight a need for a tumor model that more accurately reflects human tumor complexity and is adaptable for high-throughput applications. Preliminary studies indicate a high correlation in drug sensitivity between tumor organoid models and corresponding patient samples in preclinical settings [38–40]. Moreover, these models allow for the simultaneous assessment of drug effects on tumor cells and normal renal tissues, facilitating the identification of anti-tumor drugs that selectively target tumor cells while sparing healthy cells, and thus highlighting potential adverse reactions [41].

Cao et al. [42] successfully developed and validated organoids derived from a patient with metastatic translocation renal cell carcinoma (tRCC) featuring PRCC-TFE3 fusion. This groundbreaking work included a high-throughput screening (HTS) process, conducted for the first time on these organoids. Their results indicated Crizotinib as a potential targeted therapeutic agent for metastatic tRCC with PRCC-TFE3 fusion. This approach, utilizing organoids and HTS assays, holds promise as a model system in translational research, potentially accelerating the creation of clinical strategies. Grassi et al. [43] explored the response of renal cancer organoids and healthy renal organoids, derived from four patients, to traditional targeted therapies sunitinib and temsilimus over 72 h. Their observations from the RCC organoids revealed that neither drug significantly affected phosphorylation of mammalian target of rapamycin (mTOR), vascular endothelial growth factor receptor 2 phosphorylated at Y996 residues, or extracellular regulated protein kinase phosphorylated at T202/Y204 residues, nor did they alter CyclinD1 expression levels, showing the effectiveness of the drug.

Organoids derived from RCC present significant opportunities in the screening and evaluation of new therapeutic agents. By integrating novel drugs into the culture medium of kidney cancer organoids, their suppressive effects on tumor cells and potential toxicities to normal cells can be methodically assessed. These RCC organoids are also instrumental in determining the specificity of new drugs towards kidney cancer cells. Through investigating the response of renal cancer cells to these new agents, their specific targets and mechanisms of action can be elucidated. Therefore, RCC organoids are invaluable in pharmaceutical research and development, offering critical insights for the advancement of novel drug therapies.

### Understanding the tumor microenvironment

The tumor microenvironment is a complex array, encompassing not only the surrounding extracellular matrix but also a diversity of cell types, each playing a vital role in the functionality of organoids. These organoids are increasingly being recognized as crucial tools for exploring how microenvironment interactions influence cellular behavior in RCC. Traditionally, oncological research has largely focused on the tumor’s epithelial aspect, considering carcinomas are epithelial in origin [44]. However, recent insights have highlighted that cancer involves a multifaceted interplay of aberrant interactions among malignant cells within the stromal compartment. This paradigm shift is reshaping therapeutic strategies, emphasizing the role of cancer-associated stromal cells. These cells are known for secreting a variety of growth factors, cytokines, and chemokines, significantly contributing to tumor growth and advancement. Organoids derived from RCC cell lines have effectively mirrored the invasive traits seen in original tumors [45]. Yet, it remains essential to verify the extent to which these cells accurately represent their tumors of origin.

In their study, Neal et al. [16] applied the ALI method to culture RCC tissues directly from patients. Utilizing advanced techniques such as immunofluorescence, flow cytometry, and single-cell sequencing, they verified that the resulting ALI-PDOs maintained the composition of the tumor microenvironment from the original tumors, including critical elements like fibroblast matrices and immune cell populations. Importantly, these organoids, with intact immune components, were used to model the effects of immunotherapy in vitro. For example, the introduction of nivolumab into the organoids stimulated T cell activity, leading to cytotoxicity against the tumor cells. This setup provided an innovative platform to assess the responsiveness of patient-derived organoids to immune checkpoint inhibitors, marking a significant step forward in personalized immunotherapy development. The integration of organoid co-culture models that replicate the tumor microenvironment is instrumental in enhancing research on tumor immune evasion and immunotherapy.

### Biomarkers identification

Renal cancer organoids have demonstrated a remarkable ability to mirror the histological and molecular traits of their originating tumors. Consequently, these organoids are gaining recognition as valuable assets in the discovery of biomarkers and the crafting of tailored therapies for renal cancer patients.

Biomarkers are pivotal molecules that provide insights into the presence, progression, and therapeutic response of carcinomas. The examination of various biological markers enables a comprehensive understanding of a patient’s specific condition, paving the way for the formulation of customized therapeutic approaches.

In their groundbreaking research, Fendler and team utilized cancer stem cells from tumors to create 3D organoids. They conducted genome-wide expression analysis on FAC-sorted CXCR4 + MET + CD44 + cells, sphere cells, and non-sorted control cells from the tumors of three individuals. This analysis uncovered the WNT/NOTCH signaling pathway as a potential focal point for customized therapies. Subsequently, inhibitors targeting WNT and NOTCH pathways were applied to these 3D organoids to evaluate their specific therapeutic impacts [50]. This study underscores the utility of organoids in identifying certain biomarkers, thereby laying the groundwork for personalized treatment approaches.

In their innovative study, Kondo et al. [46] utilized renal cancer organoids to discover a potential biomarker for renal cell carcinoma. They observed that the expression level of the protein CD109 was markedly elevated in renal cancer organoids compared to normal kidney organoids. Further, they established a correlation between high CD109 expression and poor prognosis in renal cell carcinoma patients. These insights indicate that CD109 could serve as a valuable biomarker for identifying individuals at increased risk for renal cell carcinoma to help diagnosis, and could also aid in the development of personalized treatment strategies for this condition.

### Assessment of Chimeric Antigen Receptor (CAR)-mediated cytotoxicity

CAR-mediated cytotoxicity involves engineering immune cells to identify and eradicate cancer cells [47]. This strategy has shown considerable promise in treating a variety of cancers, including RCC [48]. Recent advancements have led to the development of innovative platforms for examining the effectiveness of CAR cells against 3D patient-derived organoids [49]. These platforms facilitate detailed observation of effector cell recruitment and cytolytic action at an individual organoid level, offering critical insights into the intricacies of CAR-mediated cytotoxic mechanisms.

While CAR-T therapy shows immense promise in RCC treatment, the need for a practical and effective method to evaluate its patient-specific efficacy remains. Renal cancer organoids have emerged as an essential tool in this regard. In a notable study, Zhichao Li and colleagues [18] leveraged patient-derived organoids to test the effectiveness and tumor selectivity of CD70-specific CAR-T cells. Their research demonstrated that these patient-derived organoids could effectively serve as a preclinical platform to gauge both the efficacy and tumor specificity of CAR-T cell therapies.

### Personalized treatment

The intrinsic heterogeneity of cancer markedly affects its tendencies for metastasis, recurrence, and resistance to drugs. Hence, it is imperative to pursue personalized treatment approaches for RCC, underscoring the need to establish tumor models that mirror the unique characteristics of each individual patient’s cancer [50].

PDX models have emerged as a sophisticated method in personalized treatment prediction, preserving a majority of the original tumor’s characteristics at histopathological, molecular, and genetic levels, thereby providing a high degree of representation for the primary tumor. These models demonstrate a strong correlation between the outcomes of in vitro drug sensitivity tests and the clinical responses of patients. However, the drawbacks of PDX models, such as the extensive time span (6–8 months) and significant costs involved in xenotransplantation, along with the potential for immune rejection, limit their extensive use [51, 52]. In contrast, Patient-Derived Organoids (PDOs), which are also sourced from patients, are 3D in vitro cultured tumor models. These models eliminate the necessity for tumor transplantation onto immunodeficient mice across different species, effectively avoiding these limitations. PDOs facilitate the development of optimal drug treatment regimens to minimize adverse drug reactions and reduce the recurrence of tumors. Additionally, the diverse genetic mutations in patient populations emphasize the importance of using PDOs molecular genotypic analysis as a key method for guiding patient treatment [53].

In a recent study, fresh tissue samples from 42 patients who had undergone nephrectomy for renal cell carcinoma were procured. These tissues were finely chopped and then cultured using a collagen-based 3D ALI cultivation system. The resultant organoids, termed ALI-PDOs, were subjected to immunohistochemical analysis and RNA sequencing. Additionally, a subset of 10 ALI-PDOs were treated with cabozantinib, a drug known for its specificity in targeting cancer cells. Observations indicated that the ALI-PDOs exhibited diverse responses to this treatment, highlighting the potential of the ALI-PDO approach in providing insights into individual patient responses to specific treatments [17].

Utilizing organoid culture technology, it is feasible for each RCC patient to establish a personalized tumor organoid repository. This biobank enables the carrying out of dosage trials with various oncological medications, in conjunction with comprehensive whole genome and transcriptome sequencing analyses. Such an approach facilitates the examination of the relationship between the genomic characteristics of different patients and their responses to drug sensitivity tests. Consequently, a unique drug sensitivity profile for each patient can be developed, offering valuable preclinical data that can inform and guide tailored treatment strategies. And with the short culture time of organoids, which can accurately reflect the characteristics of individual patient tumors, organoids have shown high clinical utility in personalized treatment .

## Limitations

While organoid models in tumor research display considerable promise, several challenges persist. Primarily, most organoids derived from normal or tumor tissues are of epithelial origin, with scant attention paid to tumors of non-epithelial origin. Additionally, the success rate and proliferation efficiency in creating organoids for various tumor subtypes remain low and unpredictable. The need for enhanced methods and components in organoid development is critical for facilitating high-throughput analysis, aiming to reduce the time and cost associated with their culture. Although some in vitro co-culture systems have been developed to incorporate tumor microenvironment cells like immune cells and fibroblasts into PDOs, advancements are needed to include additional non-epithelial cell types in co-cultures [54]. Existing co-culture systems often face limitations in culture duration, and establishing long-term co-culture systems presents a significant challenge. Furthermore, the role of the extracellular matrix in influencing PDO phenotypes and drug responsiveness is not fully understood, and there is a lack of suitable 3D culture platforms to accurately mimic these interactions among ECM.

### Difficulties in establishing the organoid models

#### Lack of clear biomarkers

Currently, there is no standardized approach for biomarker sampling or analysis in renal cancer research, presenting unique challenges. For instance, blood-based biomarkers are prone to degradation by various enzymes like proteases and nucleases. Unlike certain cancers such as breast cancer, which have well-established molecular markers, RCC lacks a universally recognized molecular marker. High-throughput microarray analysis has been employed in the past to delineate the molecular markers of each histological subtype of RCC. However, the utility of gene expression profiles has predominantly been confined to ccRCC, focusing on identifying its characteristic mutations, alterations, and biomarkers through genome-wide studies [55].

RCC is characterized by its genomic diversity, with distinct mutations varying according to histological subtypes. There are four primary histological subtypes of renal cell carcinoma [56], each exhibiting unique molecular and clinical features. This diversity, especially evident in the ccRCC subtype, includes specific genetic alterations such as the loss of VHL tumor suppressor gene function, inactivation of SET domain-containing protein 2 (SETD2), KDM6A, KDM5C (lysine-specific demethylase), and polybromo-1 (PBRM1). Moreover, the papillary RCC (PRCC) subtype is known for its specific proto-oncogene: MET. Though organoids can help find biomarkers as mentioned above, the continuous discovery of new subtypes adds complexity to the accurate diagnosis of renal cancer subtypes, posing challenges to renal cancer biology study and drug development [57].

#### Heterogeneity of inherited genetic mutations

Recent studies highlight the significant role of heterogeneity in renal cancer. Intratumoral heterogeneity, which manifests as genetic variations among cells within the same tumor, has been a subject of study for many years. This diversity arises each time a cell divides, accruing new mutations, making it highly unlikely to find two genetically identical cells within a single tumor. Consequently, genetic heterogeneity becomes a constant presence, impacting patient responses to treatments. Therefore, a comprehensive approach involving whole genome analysis of both the individual’s germline genome and the tumor itself is essential. Despite continuous efforts in RCC biomarker research, the challenge of designing unique drugs for every patient remains formidable. Thus, the inherent heterogeneity of RCC may substantially limit the effectiveness of biomarker-driven studies [58].

#### Inefficient culture

Organoids have emerged as crucial in both biological research and clinical decision-making for treating renal cancer, yet the limitations and challenges of this approach are not to be underestimated. Kidney organoids have been successfully derived from various sources, such as hPSCs, adult or fetal kidney tissues, and renal cancer biopsies. Despite their potential, organoids from adult kidney and renal cancer are still at a nascent stage, particularly for applications like drug screening and nephrotoxicity assessment. Organoids from adult stem cells could better mirror patient-specific conditions [19]. The success rate for developing organoid cell lines from metastatic biopsy tissues is around 15–20%. Additionally, creating renewable primary renal cancer cell lines is a complex task due to the dominance of nonmalignant cells in the samples.

### Lack of microenvironment

Current organoid cultures, primarily based on epithelial cells, fail to replicate the complex tumor microenvironment present in the human body. This limitation is due to the absence of essential cellular components like stroma, vascular endothelium, and immune cells [49]. Organoids, in their present form, are a simplified model, unable to capture the intricacies of the biological microenvironment akin to that of human cells. This technological limitation extends to the uncertainty in replicating the exact developmental pathways of renal organoids in vitro as compared to in vivo processes. ScRNA-seq investigations reveal a significant shortfall in disease-specific functional proteins in hPSC-derived organoids. This finding indicates a marked difference between their real-world utility and their theoretical potential [59]. In renal cancer studies, the microenvironment, including cell type variability, plays a pivotal role. The development of RCC organoid models has focused on immune markers and immune suppression, which are key targets for treatment of renal cancer. However, the interaction between cancerous epithelial cells and the peripheral immune system are ignored. Future research will necessitate incorporating additional cell types from the tumor microenvironment, such as immune cells, stromal cells, or neural cells, into organoid cultures. This approach aims to create a more accurate representation of the tumor environment [60].

### Diversity between patients

Organoids derived from renal cancer offer a novel approach for evaluating the efficacy of drugs in a manner tailored to individual patients. Among the limited number of pharmaceuticals assessed, not every drug elicited a response in the organoids that mirrored that of the primary tumor. The determination of patient-specific responses to targeted therapies in the context of early RCC diagnosis and treatment presents a notable complexity. Selecting an appropriate drug is challenging due to varying clinical benefits and drug tolerances observed among patients. Enhancing patient outcomes through targeted drug selection is a significant hurdle, necessitating the development of more precise biomarkers and predictive models. The expanding understanding of molecular subtypes of RCC through next-generation sequencing is a critical step in identifying RCC-specific genomic markers. This knowledge is instrumental in guiding treatment selection and advancing towards more sophisticated therapeutic strategies [18].

Kidney organoids, despite their inherent limitations, offer an unprecedented and distinctive model for exploring human-specific renal disease phenotypes. This innovative approach provides insights that were previously unattainable. Nevertheless, the emergence of organoid biology does not diminish the significance of traditional models. On the contrary, the coexistence of various modeling systems enables researchers to integrate findings from diverse models. Such integration fosters a more holistic understanding of renal diseases.

## Conclusion

Organoids, as 3D structures originating from stem cells or tissue-specific progenitors, demonstrate a remarkable ability to emulate the complex architecture and function of organs. Organoids also show the possibility to generate healthy tissue derived organoids, These entities have become pivotal in the study of diverse diseases, including RCC.

RCC, a primary form of adult kidney cancer originating in renal tubule cells, is notorious for its high mortality rate. Conventional 2D cell cultures fall short in replicating the kidney’s intricate 3D architecture and microenvironment, highlighting the importance of 3D organoid models in RCC research.

Organoids derived from RCC cells excel in reproducing the disease’s histological and molecular characteristics, making them invaluable in exploring RCC biology and developing new therapies. They enable the investigation of various drugs and therapies on RCC cells, including the discovery of novel therapeutic targets. Moreover, these organoids facilitate the study of interactions between RCC cells and their surrounding microenvironment, including immune and stromal cells.

In essence, the use of organoids in RCC research holds significant promise for advancing our understanding of the disease and enhancing the development of new treatments. It offers a more physiologically relevant approach to study RCC, allowing for the exploration of complex 3D structures and cell-microenvironment interactions.

## Data Availability

No datasets were generated or analysed during the current study.
